# Chromosomal imbalances are uncommon in chagasic megaesophagus

**DOI:** 10.1186/1471-230X-10-20

**Published:** 2010-02-17

**Authors:** Marilanda F Bellini, Antonio J Manzato, Ana E Silva, Marileila Varella-Garcia

**Affiliations:** 1 UNESP, São Paulo State University, Department of Biology, Campus São José do Rio Preto, SP, Brazil; 2 University of Colorado Denver, Department of Medicine/Medical Oncology, Aurora, Colorado, USA; 3 UNESP, São Paulo State University, Department of Computer Sciences and Statistics, Campus São José do Rio Preto, SP, Brazil

## Abstract

**Background:**

Chagas' disease is a human tropical parasitic illness and a subset of the chronic patients develop megaesophagus or megacolon. The esophagus dilation is known as chagasic megaesophagus (CM) and one of the severe late consequences of CM is the increased risk for esophageal carcinoma (ESCC). Based on the association between CM and ESCC, we investigated whether genes frequently showing unbalanced copy numbers in ESCC were altered in CM by fluorescence in situ (FISH) technology.

**Methods:**

A total of 50 formalin-fixed, paraffin-embedded esophageal mucosa specimens (40 from Chagas megaesophagus-CM, and 10 normal esophageal mucosa-NM) were analyzed. DNA FISH probes were tested for *FHIT*, *TP63*, *PIK3CA*, *EGFR, FGFR1*, *MYC*, *CDKN2A, YES1 *and *NCOA3 *genes, and centromeric sequences from chromosomes 3, 7 and 9.

**Results:**

No differences between superficial and basal layers of the epithelial mucosa were found, except for loss of copy number of *EGFR *in the esophageal basal layer of CM group. Mean copy number of *CDKN2A and *CEP9 and frequency of nuclei with loss of *PIK3CA *were significantly different in the CM group compared with normal mucosa and marginal levels of deletions in *TP63*, *FHIT, PIK3CA, EGFR, CDKN2A, YES *and gains at *PIK3CA, TP63, FGFR1, MYC, CDNK2A *and *NCOA3 *were detected in few CM cases, mainly with dilation grades III and IV. All changes occurred at very low levels.

**Conclusions:**

Genomic imbalances common in esophageal carcinomas are not present in chagasic megaesophagus suggesting that these features will not be effective markers for risk assessment of ESCC in patients with chagasic megaesophagus.

## Background

Chagas' disease is a human tropical parasitic disease which occurs in the Americas, particularly in South America. It affects 16 to 18 million people in tropical and subtropical countries of Latin America [1]; in Brazil, the number of cases has reached 6 million [2]. During the chronic phase, 6 to 7% of chagasic patients develop mega syndromes represented by muscular hypertrophy and dilation of the esophagus or colon, in consequence of destruction of the myoenteric and submucous plexus by the protozoan *Trypanosoma cruzi*[3]. The digestive forms, megaesophagus or megacolon may be observed in advanced stages of the disease [3].

Megaesophagus is consequence of achalasia characterized by the destruction or lack of intramural nerve plexus, which determines the absence of peristalsis and lack of openness of the lower esophageal sphincter in response to swallowing. In consequence, food retention or esophageal stasis occurs, leading to the appearance of chronic esophagitis, acanthosis, paraceratose and leukoplakia, possibly pre-cancerous lesions [3]. One of the severe late consequences of chagasic megaesophagus is the increased risk (3% to 8%) of developing esophageal squamous cell carcinoma (ESCC) compared to when megaesophagus is not present [4-6]. Also, ESCC develops in chagasic megaesophagus patients at a younger age than in those without this disease [5]. The detection of cancer in these patients is difficult because the symptoms are hidden by the severe dysphagia caused by megaesophagus [7]. The diagnosis is frequently late, when the patient is in advanced stage, resulting in a poor prognosis [5].

ESCC has been reported as the ninth most common malignancy and ranks the sixth most frequent cause of death worldwide, but its incidence varies largely among regions [8]. Approximately 16,470 new cases of esophageal carcinoma were expected in the US population in 2009 [9]. The Brazilian National Institute of Research in Cancer (INCA) reported that esophageal cancer was the sixth in the cancer rank mortality in 2000 with 5,307 deaths. The estimated incidence in 2008 was 10550 new cases with an incidence per 100 thousand individuals varying among geographical areas from 1.04 to 19.07 in males and 0.39 to 7.58 in females [8].

In ESCC, molecular cytogenetic techniques have shown common occurrence of unbalanced genomic regions involved in amplification of oncogenes and deletion of tumor suppressor genes [10,11]. However, studies in benign esophageal lesions with precancerous potential as megaesophagus are scarce. This study used FISH technique to investigate chagasic megaesophagus for genomic status of genes frequently unbalanced in ESCC with the goal of identifying potential markers of risk to cancer. The selected targets included *FHIT*, *TP63*, *PIK3CA*, *EGFR, FGFR1*, *MYC*, *CDKN2A, YES1 *and *NCOA3 *genes, all of which have been reported as occurring in significantly abnormal numbers in ESCC [12-19].

## Methods

### Subjects

Formalin-fixed, paraffin-embedded (FFPE) esophageal mucosa was obtained from 40 patients with diagnosis of chagasic megaesophagus (CM) who underwent middle and distal esophageal biopsies from 2000 to 2007 at the Hospital de Base (São José do Rio Preto, SP, Brazil). The study was approved by the Institution Research Ethical Committee (CEP) and by the Brazilian National Research Ethics Committee (CONEP), and written informed consent was obtained from all patients. Among the 40 CM patients, the mean age was 62.5 years (range: 40 - 83 years) and 20 were male; 29 were never-alcoholics, 3 former-alcoholics (≥5 years of abstinence) and 8 current alcoholics; 18 were never-smokers, 6 former-smokers (≥5 years of quitting) and 16 were current smokers. The megaesophagus diagnosis was made by physical examination, and radiologic and endoscopic study of esophageal motility by manometry [3]. The dilation grade of megaesophagus was classified as I to IV, based on the retention of contrast, diameter, tonicity of the lower sphincter and the length of the esophagus body [20]. This study cohort included 5 patients with megaesophagus grade I, 7 with grade II, 17 with grade III and 11 with grade IV. The CM group was followed for 2 to 4 years (median follow up = 2,8 years) and no one developed ESCC, probably due to the short follow up of patients.

The control group (NM) was composed by 10 health subjects who were submitted to endoscopy under suspicion of dyspepsia, but the histopathological analysis has shown normal esophageal mucosa. In this group, the mean age was 42.4 (range: 26 - 67 years); 3 individuals were male and 7 female; 6 were never-alcoholics and four current alcoholics; 7 were never smokers and 3 current smokers.

### Fluorescence In Situ Hybridization (FISH)

Serial 4 μm-thick sections were cut from paraffin-embedded blocks and mounted in glass slides pre-treated with 3-aminopropyl-triethoxysilane/acetone solution. Specimens were incubated at 56°C overnight, deparaffinized in Citrisolv washes (Fisher Brand, Cat #22-143975) (three times for 10 min each), and dehydrated in 100% ethanol. After incubation in 2 × SSC (Sodium chloride, sodium citrate solution, pH 7.0) at 75°C, sections were digested with proteinase K (0.25 mg/ml in 2 × SSC, pH 7.0) at 45°C, rinsed in 2 × SSC (pH 7.0) at room temperature for 5 min and dehydrated in an ethanol series.

The following DNA probe sets were used: (a) *EGFR*/CEP7 (Abbott Molecular, Cat. # 32-191053, *EGFR *mapped at 7p12), *P16*/CEP9 (Abbott Molecular, Cat. # 32-190078, *CDKN2A *mapped at 9p12), *c-MYC *(Abbott Molecular, Cat. # 32-190006, MYC mapped at 8q24)/homebrew *FGFR1 *(RP11-350N15, mapped at 8p12), and the homebrew probes *FHIT *(CTD-2196D15, mapped at 3p14.2)/centromere 3 (pα 3.5), *TP63 *(RP11-373I6, mapped at 3q28)/*PIK3CA *(RP11-245C23, mapped at 3q26) and *YES1 *(RP11-769O8, mapped at 18q11.31)/*NCOA3 *(RP11-456N23, mapped at 20q12). The homebrew probes were prepared from BAC clones and purified DNA was labeled using the Nick Translation Kit (Abbott Molecular Cat. # 32-801300). In each probe set, one target was labeled with green fluorophore (Spectrum Green) and the other with red fluorophore (Spectrum Red). The probe set was applied to the selected area, which was covered with glass coverslip and sealed with rubber cement. Co-denaturation of chromosomal and probe DNAs was performed at 85°C for 10 min and hybridization was allowed to occur in a humidified chamber at 37°C for 20-24 h for commercial probes and 40-48 h for homebrew probes. After hybridization, the slides were washed twice in 2 × SSC/0.3% NP-40 at 73°C for 2 min, rinsed in 2 × SSC at room temperature for 2 min, dehydrated in ethanol series, air dried and counterstained with 4', 6'-diamino-2-phenylindole - DAPI, (0.3 ug/ml in Vectashield Mounting Medium, Vector, Cat. # H-1200).

Fluorescence signals were scored in epifluorescence microscope using single band filters for DAPI, FITC (fluorescein), and Texas red, double-band pass filter (FITC and Texas red) and triple-band pass filter (DAPI, FITC, and Texas Red). Histological areas previously selected in the HE-stained (hematoxylin and eosin) sections were identified in the hybridized slides. Signals were scored in 100 epithelial nuclei per specimen, in at least four distinct areas: 50 nuclei in the superficial layer (larger cells, closer to the lumen) and 50 nuclei in the basal layer (darker HE-stained nuclei, smaller cells, closer to the muscular layer). The scoring was performed in both layers to check for differences between proliferate activity, since basal layer cells are in high proliferative activity, whereas the superficial layer cells are more mature. For 3 genes, *FHIT*, *EGFR* and *CDKN2A*, the experiments were performed including centromeric sequences of carrier chromosomes as an internal control, respectively chromosomes 3, 7 and 9. This design was necessary for *FHIT* and *CDKN2A* since these genes are known for their loss in cancer specimens [21,22]. For each FISH probe set, tissue sections from two normal subjects were used as control.

### Statistical Analysis

Descriptive statistics were calculated using an Microsoft Excel macro template previously validated including mean copy number per cell, standard deviation and frequency of cells with 1, 2 and ≥3 copies of each DNA target tested, as well as the ratio for gene/control probe when applicable. The superficial and basal layers were compared by *t*-student paired test, and between the experimental groups were compared by *t*-student unpaired test; in both cases confidence level was established as 0.05 [23]. Association between gene status with age, gender, life style factors (smoking and alcoholism), and megaesophagus grade in the CM group was compared by ANOVA and the comparisons for losses and gains, and aneusomies, between the groups, were done by χ^2 ^test. One exploratory analysis using three-dimensional plots was performed for the percentages of genes status classes.

## Results

The descriptive indexes (mean and standard deviation) for the tested genes (*FHIT*, *PIK3CA, TP63*, *EGFR*, *FGFR1*, *MYC*, *CDKN2A, YES1*, *NCOA3*) and centromeric sequences (chromosomes 3, 7 and 9 evaluated respectively with the genes *FHIT*, *EGFR *and *CDKN2A*), and the results of the statistical analyses for comparison of mean copy numbers between the superficial and basal layers in each group are presented in Table 1. The mean values in the CM group ranged from 1.49 to 1.78 in the superficial layer and from 1.45 to 1.79 in the basal layer. In the NM group, the mean frequencies ranged from 1.35 to 1.77 in the superficial layer and from 1.61 to 1.79 in the basal layer. Superficial and basal layers of the esophageal mucosa did not show significant differences, excepted for *EGFR *in the CM group (p = 0.008) that showed lower copy number in the basal layer. Results from both layers were combined for comparison of mean copy numbers per cell of the each molecular target and no difference was detected between CM and NM. However, the copy number of *CDKN2A* was significantly lower (p < 0.0001) in the CM group compared to NM, and similar results were observed for the corresponding centromere 9 (CEP9) (p = 0.0002).

**Table 1:** Mean copy number per cell for the 12 DNA targets tested in esophageal mucosa of chagasic megaesophagus and normal mucosa groups.

	CM			NM			
		
Targets	Mucosa Layers			Mucosa Layers			*t-Student* CM × NM
					
	Superficial	Basal	Total	Superficial	Basal	Total	
		
	Mean ± SD			Mean ± SD			
*FHIT*	1.77 ± 0.08	1.78 ± 0.08	1.76 ± 0.08	1.35 ± 0.49	1.75 ± 0.04	1.74 ± 0.04	^ns ^P = 0.7803
	Range: 1.56-1.90			Range: 1.71-1.77			
*PIK3CA*	1.76 ± 0.09	1.78 ± 0.08	1.77 ± 0.07	1.70 ± 0.09	1.78 ± 0.11	1.74 ± 0.01	^ns ^P = 0.6982
	Range: 1.56-1.96			Range: 1.64-1.86			
*TP63*	1.77 ± 0.11	1.78 ± 0.09	1.77 ± 0.09	1.70 ± 0.09	1.78 ± 0.11	1.74 ± 0.01	^ns ^P = 0.5304
	Range: 1.56-2.06			Range: 1.64-1.86			
*EGFR*	1.52 ± 0.12^a^	1.45 ± 0.15^b^	1.49 ± 0.13^a, b^	1.60 ± 0.00	1.74 ± 0.01	1.67 ± 0.01	^ns^P = 0.3700
	Range: 1.25-1.80			Range: 1.60-1.75			
*FGFR1*	1.78 ± 0.01	1.76 ± 0.10	1.77 ± 0.08	1.63 ± 0.01	1.70 ± 0.03	1.67 ± 0.03	^ns ^P = 0.2200
	Range: 1.56-2.22			Range: 1.62-1.72			
*MYC*	1.76 ± 0.09	1.75 ± 0.09	1.76 ± 0.08	1.66 ± 0.01	1.74 ± 0.02	1.70 ± 0.01	^ns ^P = 0.5396
	Range: 1.58-1.77			Range: 1.69-1.70			
*CDKN2A*	1.49 ± 0.08	1.50 ± 0.07	1.50 ± 0.06	1.76 ± 0.14	1.72 ± 0.01	1.74 ± 0.07	* P < 0.0001
	Range: 1.34-1.59			Range: 1.69-1.79			
*YES1*	1.67 ± 0.13	1.67 ± 0.12	1.67 ± 0.11	1.70 ± 0.08	1.68 ± 0.13	1.69 ± 0.07	^ns ^P = 0.8099
	Range: 1.37-1.99			Range: 1.58-1.75			
*NCOA3*	1.65 ± 0.11	1.64 ± 0.11	1.64 ± 0.10	1.66 ± 0.11	1.61 ± 0.01	1.64 ± 0.06	^ns ^P = 0.7656
	Range: 1.37-1.80			Range: 1.59-1.68			
Centromere 3	1.77 ± 0.08	1.79 ± 0.07	1.77 ± 0.07	1.71 ± 0.07	1.76 ± 0.08	1.74 ± 0.08	^ns^P = 0.6638
	Range: 1.56-1.90			Range: 1.68-1.79			
Centromere 7	1.53 ± 0.12	1.50 ± 0.16	1.52 ± 0.13	1.67 ± 0.01	1.79 ± 0.04	1.73 ± 0.21	^ns^P = 0.1235
	Range: 1.26-1.53			Range: 1.76-1.82			
Centromere 9	1.53 ± 0.09	1.53 ± 0.09	1.53 ± 0.07	1.77 ± 0.15	1.77 ± 0.04	1.77 ± 0.10	* P = 0.0002
	Range: 1.33-1.65			Range: 1.70-1.84			

CM, chagasic megaesophagus; NM, normal mucosa; SD, standard-deviation; Statistical Analysis: *t*-Student; *, P < 0.05; ^ns^, P > 0.05; ^a, b^, P = 0.008.

In another investigative approach, frequencies of nuclei with normal (2 copies) or loss (<2 copies) and gain (>2 copies) for each gene were compared (Table 2), and only for *PIK3CA* there were higher frequencies of cells with copy number loss in the CM group compared to NM (36.4% vs. 28.5%). Statistical analysis was not performed for gain due to the scarcity of cells with this pattern. Aneusomies involving centromeres of chromosomes 3, 7, and 9 were also investigated (Table 2). The CM group showed significantly increased frequencies of cells with copy number loss of centromeres of chromosomes 7 and 9 (51% and 44%, respectively) in comparison to the NM group (28% and 23%, respectively). The CM group also showed numerically higher frequencies of cells with copy number gain of centromeres of chromosomes 7 and 9 (1.68% and 10.38%, respectively) than the NM group (0.50% and none, respectively), although the numbers were too small for statistical analyses.

**Table 2:** Frequencies of cells with loss, normal status and gain in copy numbers of the target in chagasic megaesophagus and normal mucosa groups.

	**<2 copies per cell**			**2 copies per cell**		**>2 copies per cell**	
			
**Target**	**CM**	**NM**	**χ**^2^**test**	**CM**	**NM**	**CM**	**NM**
			
*FHIT*							
N	20	2	P = 10.672^ns^	2478	144	6	3
%	0.79	1.34		98.96	96.64	0.24	2.04
*PIK3CA*							
N	898	57	P = 0.000*	1530	139	35	4
%	36.39	28.50		61.99	69.50	1.42	2.00
*TP63*							
N	885	58	P = 2.579^ns^	2878	136	37	6
%	23.29	29.00		75.74	68.00	0.97	3.00
*EGFR*							
N	298	12	P = 1.067^ns^	3433	187	171	1
%	7.59	6.00		88.03	93.50	4.38	0.50
*FGFR1*							
N	928	68	P = 4.172^ns^	2833	121	46	1
%	24.38	34.00		74.41	60.50	1.21	0.50
*MYC*							
N	918	62	P = 4.812^ns^	2849	135	33	2
%	24.00	31		75.00	67.50	0.01	1.00
*CDKN2A*							
N	183	6	P = 17.702 ^ns^	3649	194	68	0
%	4.69	3.00		93.56	97.00	1.74	0.00
*YES1*							
N	1381	62	P = 2.171^ns^	2407	136	12	2
%	36.34	31.00		63.34	68.00	0.32	1.00
*NCOA3*							
N	45	8	P = 3.627^ns^	2366	130	8	0
%	1.86	5.80		97.81	94.20	0.33	0.00
CEN3							
N	683	54	P = 0.470^ns^	2510	145	7	1
%	21.0	27.0		78.44	72.5	0.56	0.50
CEP7							
N	1637	56	P = 0.00+*	1509	1143	54	1
%	51.16	28.0		47.16	71.50	1.68	0.50
CEP9							
N	1400	46	P = 0.00+*	1468	154	332	0
%	43.75	23.0		45.87	77.0	10.38	0.00

CM, chagasic megaesophagus; NM, normal mucosa; SD, standard-deviation; Statistical Analysis: *t*-Student; *, P < 0.05; ^ns^, P > 0.05; ^a, b^, P = 0.008.

The gene/centromere ratios for *FHIT/*CEN3*, EGFR/*CEP7 and *CDKN2A/*CEP9, the probe sets including matched targets in the study, were all balanced (close to 1) and differences were not detected between the mean copy number of each target within each group (Table 3). These results suggest that loss of FHIT, EGFR and CDKN2A was accompanied by loss of their respective chromosomes.

**Table 3:** Comparison between matched gene and centromere targets, in chagasic megaesophagus and normal mucosa groups.

Gene-Centromere	CM		NM	
	
	Mean ± SD	Ratio	Mean ± SD	Ratio
*FHIT*	1.77 ± 0.08	1.00	1.74 ± 0.04	1.00
CEN3	1.77 ± 0.08		1.74 ± 0.08	
*t*-Student	ns		ns	
*EGFR*	1.49 ± 0.12	0.98	1.67 ± 0.01	0.96
CEP7	1.51 ± 0.13		1.73 ± 0.02	
*t*-Student	ns		ns	
*CDKN2A*	1.51 ± 0.09	1.00	1.74 ± 0.07	0.98
CEP9	1.54 ± 0.10		1.77 ± 0.10	
*t*-Student	ns		ns	

Chagasic megaesophagus, CM; normal mucosa, NM; SD, standard-deviation; Statistical Analysis: *t*-Student ns, p > 0.05.

**Figure 1:**
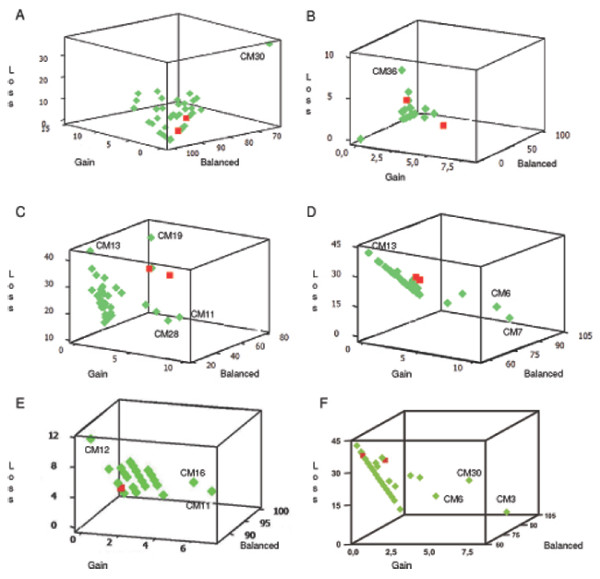
**Exploratory analysis using three-dimensional plots of the percentages of genes status for individual cases is illustrated**. Red square: Normal Mucosa; Green Square: Megaesophagus, A. *EGFR*, Mean frequency of loss in the CM = 6.84%, CM30 ~30% of loss; B. *FHIT*, Mean frequency of loss in the CM = 0.63%, CM36 ~10% of loss; C. *PIK3CA*, Mean frequency of loss: 23.63%, Mean frequency of gain: 0.92%, CM13 and CM19 ~40% of loss, CM28 and CM11, between 5 and 10% of gain; D. *TP63*, Mean frequency of loss = 23.29%, Mean frequency of gain = 1.07%, CM13 ~45% of loss, CM6 and CM7 ~10% of gain; E. *CDKN2A*, Mean frequency of loss = 4.76%, Mean frequency of gain = 1.79%, CM12 ~12% of loss, CM 11 and CM 16 ~6% of gain; F. *MYC*, Mean frequency of gain = 0.87%, CM3, CM6 and CM30 between 5 and 7.5% of gain.

**Figure 2:**
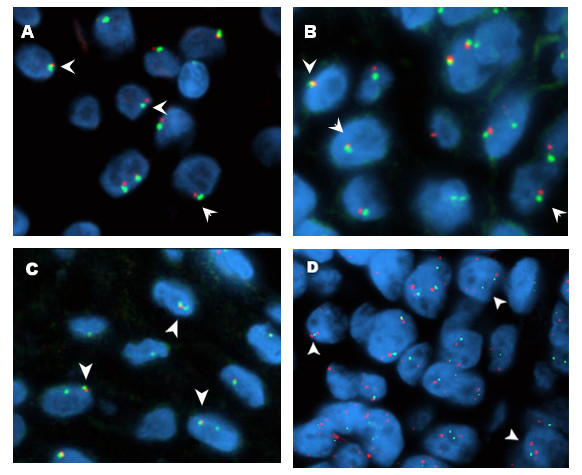
**FISH images for the unbalanced genes and centromeric targets in the megaesophagus specimens**. Dual-target, dual-color color hybridization in chagasic megaesophagus. A. Loss of *CDKN2A *(red signal) and centromere 9 (green signal); B. Loss of *EGFR *(red signal) and centromere 7 (green signal); C. Loss of *FHIT *(red signal) and centromere 3 (green signal); D. Gain of *TP63 *(red signal) and loss of *PIK3CA *(green signal). Arrows indicated cells with abnormal copy numbers of the genes.

Data were also subject to an exploratory analysis using three-dimensional plots of the percentages of cells per genomic pattern (loss, balanced and gain) for individual cases as illustrated in Figure 1. Several cases were identified harboring deletions on specifics targets: *CM7 (YES1), CM12 (CDKN2A), CM13 (PIK3CA; TP63), CM19 (PIK3CA), CM30 (EGFR), CM36 (FHIT; *CEP9*), CM16* and *CM34 (CEP9)*. Other cases showed genomic gain for specific targets: *CM3 (MYC), CM6 (TP63; MYC), CM7 (TP63), CM11 (PIK3CA; CDKN2A), CM16 (CDKN2A), CM28 (PIK3CA), CM30 (MYC; FGFR1), CM36 (NCOA3),* and *CM11* and *CM21 (*CEP9*)*. Figure 2 illustrates unbalanced status of genes and centromeric DNA targets in megaesophagus specimens.

No significant association was detected in CM group between mean copy number per cell of each DNA target tested and parameters such as age, gender and the life style factors tobacco smoking and alcoholism although the small size of each subset impaired robust conclusion. Similarly, no significant differences were observed on mean copy number per cell of each genomic target according the dilation grades of megaesophagus (data not shown). However, the CM cases identified with genomics deletions and gains in the three-dimensional plots were mostly classified as grades III and IV, suggesting that chromosomal imbalances are more likely to occur in more advanced grades.

## Discussion

Esophageal squamous cell carcinoma is one of the most prevalent cancers worldwide and has multifactorial origin, in which environment factors, especially alcohol ingestion and smoking, play significant role [10,24]. Several genetic alterations are also associated with ESCC, such as chromosomal changes, allelic deletions, activation of oncogenes and inactivation of tumor suppressor genes [10,11]. In tropical and subtropical countries of Latin America such as Brazil, the risk of ESCC may also be related to megaesophagus due to Chagas' disease. The period between onset of symptoms related to achalasia and detection of cancer ranges from 17 to 28 years [4]. In the current study no patient developed esophageal carcinoma, which can also be due to insufficient follow up.

This study showed that genomic copy number changes were not common events in megaesophagus. Among the tested 12 DNA targets mapped to 6 distinct chromosomes, there was only marginal differences between specimens from chagasic megaesophagus and normal esophageal mucosa. The only statistically significant difference in copy number between groups involved *CDKN2A*. Loss of function of *CDKN2A *(also called *MTS-1 *or *P16*) prevents the blocking of G1 phase and appears to be a necessary event for the progression of pre-cancerous cells to malignancy [25,26]. Genetic and epigenetic alterations in *CDKN2A *have been reported in early stages of esophageal carcinogenesis [27-30]. In primary esophageal carcinomas and cell lines, deletions and point mutations in *CDKN2A *gene have been reported in 16% to 82% of cases [22,31], and hypermethylation of promoter region in 20% to 88% of ESCC and precancerous lesions [30,32]. The current study detected deletion of *CDKN2A *and CEP 9 sequences in CM compared with NM group. In agreement with these findings, patients with idiopathic achalasia and chagasic megaesophagus, with or without esophageal carcinoma, showed reductions in expression of p16 protein [7] and Bellini *et al. *[33] observed a marginal decrease in p16 protein expression in chagasic megaesophagus. Additionally, recent subset analyses by our group did not find mutations in *CDKN2A *(exons 1 and 2) and *FHIT *(exons 5 and 7) genes, suggesting these events are uncommon in CM [34], and have detected copy number changes of chromosomes 7, 11 and 17 and *TP53 *deletion by FISH [35].

The tumor suppressor gene *FHIT *(3p14.2) is deregulated during the development of ESCC. Deletion in both *FHIT *alleles results in failure in the transcript [36] and consequent absence or reduction of Fhit protein, which act in the cell cycle regulation and apoptosis. Genetic and epigenetic alterations of *FHIT *are associated with development of several cancer types [37,38]. Loss of heterozygosity (LOH) in areas of the 3p14-p21 bands has also been reported in low-grade dysplasia and postulated as early events in esophageal carcinogenesis [39]. While hypermethylation of *FHIT *gene has been described in 33% to 69.4% of ESCC cases, it is also known that deletion and loss of protein expression are frequent in esophageal carcinoma [32,36]. Deletion of *FHIT *observed in a single megaesophagus specimen in the present study may associate with early onset of genetic chances in these lesions, but it is worth to note that no significant decrease in the level of Fhit protein expression have been detected in chagasic megaesophagus [33].

All other tested genes are categorized as oncogenes and have been previously shown to associate with ESCC, in which they are commonly amplified [12-19]. However, abnormal copy number patterns were only seen in few individuals in the Chagasic megasophagus cohort and involved both gains and losses. One example is *PIK3CA*, which encodes the catalytic subunit that uses ATP to phosphorylate phosphatidylinositol, a gene that is frequently amplified in ESCC [40-42]. Yen et al. [40] determined in FISH assays that *PIK3CA *was amplified in cell lines and primary tumors and detected a positive correlation between amplification and tumor size, lymph node metastasis and clinical stage. Yang et al. [41] showed that *PIK3CA *gene amplification was highly correlated with protein overexpression, supporting amplification as a major mechanism for overexpression. Nevertheless, in this study *PIK3CA* was lost rather than amplified in the CM specimens as a group while involved in marginal level of loss two specimens and gained in two other specimens.

Gene amplification was one of the basic mechanisms leading to overexpression of the *TP63 *in ESCC [18,43,44]. The amplification of *TP63*, whose protein plays in the development and maintenance of stratified epithelial tissues has been described in early stage of ESCC carcinogenesis but down-regulated in advanced [18,43]. Amplification of the membrane receptor genes *EGFR* and *FGFR1 *has been reported in various cancers including ESCC [45,46], and this phenomenon has been suggested to be a poor prognostic factor in solid tumors [47]. In ESCC, co-expression of both *aFGF *and *FGFR1 *was associated with larger tumor area and worse prognosis which suggests that the membrane receptor may promote proliferation of esophageal cancer cells in an angiogenesis-independent and autocrine manner and may contribute to rapid recurrence after esophageal resection [47].

*MYC* is a transcription factor that binds E-boxes as a heterodimer with Max in about 15% of all genes, and recruits co-activators to regulate gene transcription. *MYC *is regulated in part through mitogenic stimuli and is activated constitutively in cancer cells through gene amplification, chromosomal translocation, point mutation and mitogenic stimulation [48]. *MYC *was found to be amplified in ESCC cell lines and in primary tumors [14]. *YES1 *codes a protein with tyrosine kinase activity that has been previously associated with esophageal carcinogenesis [15,42]. Similarly, the *NCOA3* gene encoding a nuclear receptor coactivator that interacts with nuclear hormone receptors to enhance their transcriptional activator function was found frequently overexpressed and also amplified in 5 to 15% of ESCCs [49-51].

Although is well established that megaesophagus preferentially affects males between the second and fourth decades of life [3], this study has not detected relationship between copy numbers of each tested gene and parameters such as age, gender, life style factors (tobacco smoking and alcoholism) and megaesophagus grade in the CM group. Nevertheless these findings may have been impacted by the limited number of samples in each category.

Interestingly, specimens with genomics deletions and gains exhibited mostly more advanced dilation grades. Megaesophagus patients have high variety of esophageal microbiota, which consists mainly of Gram-positive anaerobic bacteria in concentrations that correlated with the degree of esophageal dilation [6,52]. Bacteria in fluid stasis can undergo reduction from nitrates to nitrites with the production of N-nitrous compounds, which has potent action in esophageal carcinogenesis [52]. High grade megaesophagus present higher dilation and esophageal stasis associated with bacterial proliferation, thus the data suggest that the level of dilation and inflammation of megaesophagus could promote genetic damages and could be related to increased risk of tumor development [4,7,53].

## Conclusion

In conclusion, among 40 chagasic megaesophagus comprehensively investigated with a panel of FISH probes, copy number gain was only displayed by three specimens for *MYC*, by one specimen for *FGFR1 *and one specimen for *NCOA3*. Loss of *FHIT *and *YES1 *was seen in one specimen and of *EGFR *in another. For the remainder genes *CDKN2A, PIK3CA *and *TP63*, from one to four specimens each showed copy number gain or loss. In every case the difference was only marginal therefore not providing strong support to a biological impact. These findings show that imbalances involving the genomic regions encompassing gene sequences relevant in esophageal carcinogenesis were not found as significant events in chagasic megaesophagus. Consequently, genomic imbalances are not promising markers for assessment of ESCC risk in chagasic megaesophagus.

## List of abbreviations

*aFGF:* endothelial cell growth factor, alpha; ANOVA: Analysis of Variance; BAC: Bacterial Artificial Chromosomes; Cat. #: Catalog number; *CDKN2A:* cyclin-dependent kinase inhibitor 2A (melanoma, p16: inhibits CDK4); CEN3: Centromere 3; CEP: Research Ethical Committee; CEP7: Centromere 7; CEP9: Centromere 9; CM: patients with histologic diagnosis of chagasic megaesophagus; CONEP: Brazilian National Research Ethics Committee; DAPI: 4', 6'-diamino-2-phenylindole; *EGFR:* epidermal growth factor receptor [erythroblastic leukemia viral (v-erb-b) oncogene homolog, avian]); ESCC: Esophageal squamous cell carcinoma; FFPE: formalin-fixed, paraffin-embedded; *FGFR1:* fibroblast growth factor receptor 1; *FHIT:* fragile histidine triad gene; FISH: Fluorescence In Situ Hybridization; FITC: fluorescein isothiocyanate; HE: Hematoxylin and Eosin stain; LOH: loss of heterozygosity; *MYC:* v-myc myelocytomatosis viral oncogene homolog (avian); *NCOA3:* nuclear receptor coactivator 3; NM: health individuals with histologically normal esophagus; *PIK3CA:* phosphoinositide-3-kinase, catalytic, alpha polypeptide; Q-PCR: Quantitative - Polymerase Chain Reaction; SSC: Sodium chloride, sodium citrate solution; *TP63:* tumor protein p63; Vs: versus; *YES1:* v-yes-1 Yamaguchi sarcoma viral oncogene homolog 1.

## Competing interests

The authors declare that they have no competing interests.

## Authors' contributions

MFB carried out the probe development, molecular cytogenetic studies and drafted the manuscript. AJM performed the statistical analysis. AES and MVG conceived the study, participated in its design and execution and contributed to the manuscript. All authors read and approved the final manuscript.

## Pre-publication history

The pre-publication history for this paper can be accessed here:

http://www.biomedcentral.com/1471-230X/10/20/prepub
